# Leadership and job satisfaction among physicians in the Cyprus public healthcare system

**DOI:** 10.1186/s12913-025-13241-3

**Published:** 2025-08-06

**Authors:** Ioanna Gregoriou, Eleftheria C. Economidou, Demetris Avraam, Elpidoforos S. Soteriades, Evridiki Papastavrou, Andreas Charalambous, Antonis Stylianides, Anastasios Merkouris

**Affiliations:** 1 Department of Nursing, School of Health Sciences, Cyprus University of Technology, 30 Archbishop Kyprianos Street, Limassol, 3036 Cyprus; 2 Department of Pediatrics, Larnaca General Hospital, Larnaca, Cyprus; 3 Department of Public Health, University of Copenhagen, Copenhagen, Denmark; 4 Healthcare Management Program, School of Economics and Management, Open University of Cyprus, Nicosia, Cyprus; 5 Environmental and Occupational Medicine and Epidemiology (EOME), Department of Environmental Health, Harvard T.H. Chan School of Public Health, Boston, USA; 6 University of Turku, Turku, Finland

**Keywords:** Physicians’ leadership, Job satisfaction, Health professionals, Public healthcare system, Cyprus

## Abstract

**Introduction:**

Leadership and job satisfaction constitute important characteristics of health professionals’ employment status. We evaluated the association between physicians’ leadership and job satisfaction among health professionals in the public health sector of Cyprus.

**Methods:**

A cross-sectional survey with self-administered questionnaires was implemented among all physicians from the public health sector of Cyprus (Ministry of Health, administration offices, public hospitals and healthcare centers). In this context we used two standardized internationally validated instruments: the Multifactor Leadership Questionnaire (MLQ-5X), and the Job Satisfaction Survey (JSS).

**Results:**

A total of 690 eligible physicians were invited to participate, of whom 511 completed the survey and 9 were excluded. A final sample of 502 physicians (262 male and 235 female) were included in the statistical analyses. The mean participants’ age was 47.7 years (SD = 9.2), whilst the mean number of years of experience in the public sector was 12.4 years (SD = 8.4). Transactional and transformational leadership were strongly associated with six out of the nine job satisfaction subscales, as well as with the total job satisfaction scale. Passive leadership was negatively associated with job satisfaction. Overall, total leadership showed a strong statistically significant association with total job satisfaction [odds ratio = 3.88, 95% CI (2.27, 6.63)].

**Conclusion:**

We found strong and statistically significant associations between transactional and transformational leadership styles and health professionals’ job satisfaction in most of the job satisfaction subscales. Establishing a causal relationship between the above requires further investigations with appropriate study design.

**Supplementary Information:**

The online version contains supplementary material available at 10.1186/s12913-025-13241-3.

## Introduction

The word leadership derives from an old English word “laedere”– the one who leads, its noun “laedan”– to guide or to bring forth. The meaning of leadership has occupied human mind since ancient times, as it is found in the works of Plato, Plutarch and other ancient historians and philosophers [1, 2]. It is also one of the most studied social science phenomena. The word leadership (in Greek “ηγεσία”) comes from the ancient Greek verb “ηγέοµαι”, which means to advance, precede, lead, direct, show the way to someone [3]. The concept of leadership has a multidimensional character, while it is further broadened by the various characteristics that define a leader. In particular, leadership in the healthcare sector, constitutes one of the most vital factors within the realm of human resource management concerning health professionals. Thus, effective leadership and adoption of an appropriate tool to assess leadership in an organization becomes a crucial scientific inquiry [4]. Health professionals in general and physicians in particular constitute an important human capital for healthcare systems [5].

In parallel, employees’ job satisfaction is seen as one of the most defining factors of organizational effectiveness [6]. Employees in healthcare institutions confront many challenges daily, resulting in job dissatisfaction, which has been linked to stress [7–9], medical errors, intention to leave [10, 11], reduction in quality of care, increased medical care costs, reduced patient compliance with medical advice, and professional burnout [12–14]. Considering the importance of healthcare quality, physicians should be facilitated to “have a voice” regarding their professional duties and experiences, a feeling of being respected and valued within an organization that is part of a leadership agenda and could improve job satisfaction [15].

Several studies have examined the association of leadership with job satisfaction in healthcare workers either through systematic reviews of the scientific literature or original research studies [16–18]. The theoretical background regarding the association under study has been explored in a previous publication [19]. In a study among in Sweden, researchers showed that a favourable leadership climate and a supportive social environment among colleagues were associated with reduced levels of discontent with their current job among physicians in Swedish primary healthcare [20]. Another study from Oman showed that having a clinical postgraduate degree, a senior level of responsibility and a perception of a good inter-professional relationship, were associated with higher job satisfaction rates. The overall job satisfaction rates were higher for the quality of care and ease of practice, but lower for the relationship with leadership [21]. In addition, in a study from Canada, scientists utilized a multivariable model to show that job satisfaction among academic family medicine faculty members was a multi-dimensional construct that included five independent predictors of job satisfaction including personal accomplishment, emotional exhaustion, place of origin, overall quality of mentorship received and the rating of teamwork implying leadership contribution [22]. On the contrary, a multicenter intervention (IMPROVEjob) in Germany to improve leadership and associated outcomes including job satisfaction among German general practitioners and practice personnel was not successful in achieving its objective however, this could be partly attributed to the fact that it was implemented during the recent pandemic [23].

Our study aim was to evaluate the association between leadership and job satisfaction among physicians in the public healthcare system of Cyprus.

## Methods

The study was implemented among all physicians who were working for the public healthcare system in Cyprus based on a cross-sectional study design using a quantitative survey with self-administered questionnaires. Further details regarding the study setting have been previously published [19]. The study was approved by the Cyprus National Bioethics Committee (Protocol number ΕΕΒΚ ΕΠ 2016.02.57) and relevant permissions to conduct the survey were also obtained from the office of the Commissioner for the Protection of Personal Data and the Ministry of Health.

### Study sample

All physicians who were working in the public healthcare system of Cyprus (census) in September 2016 were invited to participate. Of those, 511 completed the survey and 9 were excluded (incomplete questionnaires). Therefore, a total of 502 physicians were included in our study.

### Data collection

The survey included four different battery tools through which we collected information on physicians’ demographics, on organizational commitment, the Multifactor Leadership Questionnaire (MLQ-5X) [24, 25] and the Job Satisfaction Survey (JSS) questionnaire developed by Spector [26]. Further details on all data battery tools have been presented previously [19].

Initially known as the MLQ-5X, its strength depends on its ability to capture several leadership styles in a single measurement instrument which is comprised of 45 items; the first 36 items measure the type of leadership style and the next 9 items the leadership outcomes. The first 36 items are used to estimate nine subscales which then determine three leadership styles: (a) transformational leadership is defined as the average of five subscales: idealized influence (attributed), idealized influence (behaviour), inspirational motivation, intellectual stimulation, and individualized consideration; (b) transactional leadership is defined as the average of two subscales: contingent reward, and management-by-exception (active); and (c) passive leadership is defined as the average of two subscales: management-by-exception (passive) and laissez-faire. The last 9 items of the questionnaire are used to estimate three subscales which consider leadership outcomes: extra effort, effectiveness, and satisfaction. Each of the 45 items is rated using a 5-point Likert scale including not at all, occasionally, sometimes, often, and frequently if not always. The score for every item ranges from 0 (not at all) to 4 (frequently if not always). Confirmatory factor analysis was used to examine the construct validity of the MLQ questionnaire. In addition, the reliability of the scale was measured through the Cronbach’s alpha formula.

The Job Satisfaction Survey is a 36 item, nine facet scale to assess employee attitudes about the job and aspects of the job. Each of the 36 items is rated using a 6-point Likert scale that ranges from 1 (‘strongly disagree’) to 6 (‘strongly agree’), while items are written in both directions (positive and negative), therefore, about half had to be reverse scored. High scores on the scale represent job satisfaction, so the scores on the negatively worded items have to be reversed before summing with the positively worded into facet or total scores. A score of 6 representing strongest agreement with a negatively worded item is considered equivalent to a score of 1 representing strongest disagreement on a positively worded item, allowing them to be combined meaningfully. The nine facets were created based on the summary score of 4 items each. These include pay, promotion, supervision, fringe benefits, contingent rewards (performance-based rewards), operating procedures (required rules and procedures), coworkers, nature of work, and communication. Scores on each of the nine facet subscales can therefore range from 4 to 24; while scores for total job satisfaction, based on the sum of all 36 items, can range from 36 to 216.

### Statistical analyses

For statistical analysis, we examined the reliability of the MLQ using Cronbach’s alpha formula. Cronbach’s alphas were computed in R through the ltm (Latent Trait Models under Item Response Theory) package, version 1.2-0. Confirmatory factor analysis (CFA) was conducted to measure whether the data from this study confirm the structural validity of the Greek version of the MLQ. The CFA was performed in R through the lavaan (Latent Variable Analysis) package, version 0.6–12, using the maximum likelihood estimation method. Two factor models were tested in this study. First, one general factor model (global leadership) in which all items load on the same factor. Second, a three-correlated factor model (transformational, transactional, and passive leadership) in which the five subscales measuring transformational leadership load on the first factor, the three subscales measuring transactional leadership load on the second factor, and the one scale measuring passive leadership load on the third factor. The relative fit of each model was assessed by different goodness of fit indices: (a) the ratio of chi-square (χ²) to degrees of freedom (df), (b) the comparative fit index (CFI), (c) the root mean square error of approximation (RMSEA), and (d) the Akaike Information Criterion (AIC).

In addition, we dichotomised each satisfaction subscale where values ​​equal or above 3.5 (i.e. the middle value of the continuous range 1–6) were considered as “job satisfaction” and values ​​below 3.5 as “not being satisfied”. Number of cases with percentages and Pearson’s Chi-squared tests with Yates’ continuity correction were used for each pair between a categorical demographic characteristic of physicians and the total job satisfaction indicator, while means with standard deviations and Mann-Whitney (i.e. two-sample Wilcoxon rank-sum) tests were used for each pair between a continuous demographic and the total job satisfaction indicator. Welch two sample t-tests were used to indicate if there is a statistically significant difference on the average scores of leadership styles between the physicians who were not satisfied by their job and those who were satisfied. Furthermore, univariable logistic regressions were performed between each leadership subscale (independent variable) and binary indicator for each job satisfaction subscale (dependent variable) to investigate their associations. We also run four additional logistic regression models examining the association between the total satisfaction (as the dependent variable) and four scales of leadership as the independent variable (transactional, transformational, passive, total). The regression models were adjusted by four potential confounding factors namely age, gender, education and workplace. We decided to avoid using the different scales of leadership in the same model to prevent any potential bias from collinearity (Table 1S– Supplement). Statistical significance was considered at a p-value of less than 5% at both sides.

## Results

A total of 502 physicians were included in the final statistical analyses. About half of the physicians were females (*n* = 262, 52.2%). The mean age of study participants was 47.7 years (SD = 9.2), whilst the mean year of experience in the public sector was 12.4 years (SD = 8.4) and in private was 4.4 years (SD = 5.9), respectively. From the 502 physicians, 69.7% were working in public hospitals and 30.3% in healthcare centers.

Cronbach’s α were calculated to assess the reliability for the 12 multi-item scales (nine for the transformational, transactional, and laissez-faire leadership styles and three for the leadership outcomes) and the three multi-scale factors. In total, 10 scales showed a good or even very good reliability in relation to Cronbach’s α (α ≥ 0.7). There were only two scales with low α: “idealized influence (attributed)”, and “contingent reward”. Also, the three factors showed good reliability with a total reliability coefficient of 0.95.

In Table 1 we present the demographic characteristics of physicians in association with job satisfaction. One chi-squared test from all pairs between the dichotomous demographic variables and the dichotomous total job satisfaction indicator, had a p-value lower than 0.05, indicating the statistically significant relationship between the variable representing physicians’ educational level and job satisfaction. In addition, all Mann-Whitney tests had a *p*-value greater than 0.05 indicating that there was no statistically significant difference in the means of age, years of experience in public health and years of experience in the private sector with respect to job satisfaction.

**Table 1:** Demographic characteristics of physicians relative to job satisfaction

Physicians’ demographics		*Total*		Total Job Satisfaction					
		*n (%)*		not Satisfied *n* (%)			Satisfied *n* (%)		*p*-value ^a^
Gender	Female	262 (52.2)		206 (51.4)			56 (55.4)		0.533
	Male	235 (46.8)		191 (47.6)			44 (43.6)		
	Missing	5 (1.0)		4 (1.0)			1 (1.0)		
Family status	Live alone	76 (15.1)		58 (14.5)			18 (17.8)		0.618
	I do not live alone	393 (78.3)		313 (78.1)			80 (79.2)		
	Missing	33 (6.6)		30 (7.5)			3 (3.0)		
Children	Yes	391 (77.9)		313 (78.1)			78 (77.2)		0.519
	No	84 (16.7)		64 (16.0)			20 (19.8)		
	Missing	27 (5.4)		24 (6.0)			3 (3.0)		
Workplace	Public Hospitals	350 (69.7)		276 (68.8)			74 (73.3)		0.455
	Health Centers	152 (30.3)		125 (31.2)			27 (26.7)		
	Missing	0 (0)		0 (0)			0 (0)		
Job position	Internal medicine	345 (68.7)		274 (68.3)			71 (70.3)		1
	Surgical	80 (15.9)		64 (16.0)			16 (15.8)		
	Missing	77 (15.3)		63 (15.7)			14 (13.9)		
Master degree	Yes	205 (40.8)		177 (44.1)			28 (27.7)		0.002
	No	240 (47.8)		178 (44.4)			62 (61.4)		
	Missing	57 (11.4)		46 (11.5)			11 (10.9)		
PhD	Yes	83 (16.5)		72 (18.0)			11 (10.9)		0.113
	No	359 (71.5)		281 (70.1)			78 (77.2)		
	Missing	60 (12.0)		48 (12.0)			12 (11.9)		
Tenure	Yes	273 (54.4)		223 (55.6)			50 (49.5)		0.284
	No	193 (38.4)		149 (37.2)			44 (43.6)		
	Missing	36 (7.2)		29 (7.2)			7 (6.9)		
Contract	Yes	193 (38.4)		148 (36.9)			45 (44.6)		0.192
	No	273 (54.4)		224 (55.9)			49 (48.5)		
	Missing	36 (7.2)		29 (7.2)			7 (6.9)		
		*n*	Mean (SD)	*n*	Mean (SD)		*n*	Mean (SD)	*p*-value ^b^
Age		471	47.7 (9.2)	372	47.7 (8.8)		99	47.7 (10.3)	0.983
Years of experience in public health		462	12.4 (8.4)	365	12.6 (8.2)		97	11.7 (9.3)	0.140
Years of experience in private		408	4.4 (5.9)	322	4.2 (5.6)		86	5.2 (7.1)	0.905

^a^Chi-squared test

^b^Mann-Whitney test

Leadership as evaluated by the Multifactor Leadership Questionnaire (MLQ-5X) relative to job satisfaction is presented in Table 2. All two-sample t-tests had a p-value less than 0.05 indicating that there was a statistically significant difference in the means of all leadership subscales between the physicians who were not satisfied by their job and those who were satisfied. Furthermore, in Table 2 we show the means of transformational, transactional, passive and total leaderships which are statistically significantly different between the physicians who were not satisfied by their job and those who were satisfied.

**Table 2:** Association of leadership with total job satisfaction

MLQ Scale	Total Job Satisfaction		*p*-value
	not Satisfied mean (SD)	Satisfied mean (SD)	
idealized influence (attributed)	1.7 (0.9)	2.4 (0.8)	< 0.001
idealized influence (behavior)	1.7 (0.9)	2.3 (0.7)	< 0.001
inspirational motivation	1.7 (0.9)	2.4 (0.9)	< 0.001
intellectual stimulation	1.5 (0.8)	2.1 (0.7)	< 0.001
individual consideration	1.5 (0.8)	1.9 (0.7)	< 0.001
contingent reward	1.3 (0.8)	2.0 (0.9)	< 0.001
management by exception (active)	1.9 (0.8)	2.2 (0.7)	0.005
management by exception (passive)	2.5 (0.9)	1.9 (0.9)	< 0.001
laissez-faire leadership	2.3 (1.0)	1.6 (0.9)	< 0.001
extra effort	1.3 (1.0)	2.1 (1.0)	< 0.001
Effectiveness	1.5 (1.0)	2.4 (1.0)	< 0.001
Satisfaction	1.6 (1.0)	2.4 (1.0)	< 0.001
Factor			
transformational leadership	1.6 (0.7)	2.2 (0.7)	< 0.001
transactional leadership	1.6 (0.7)	2.1 (0.7)	< 0.001
non-leadership	2.4 (0.9)	1.7 (0.8)	< 0.001
Total	1.8 (0.4)	2.1 (0.5)	< 0.001

**Fig. 1:**
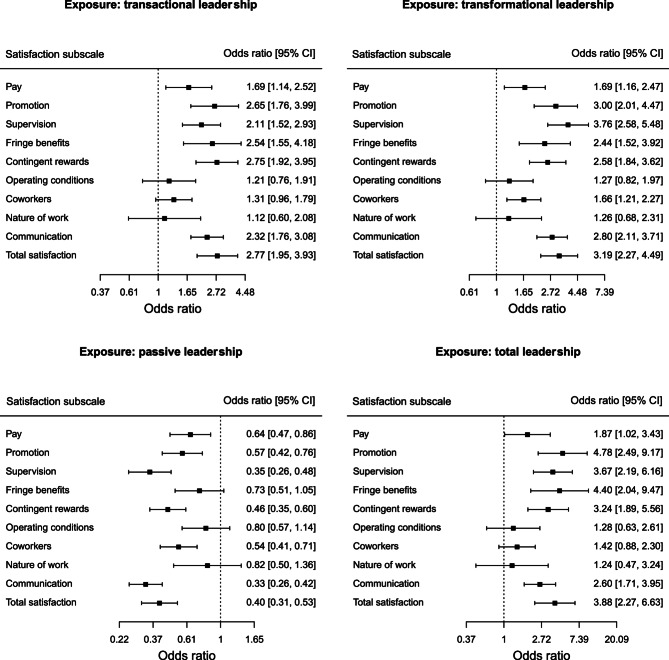
Odds ratios with 95% confidence intervals from logistic regression models for each pair between a leadership style (independent variable) and a job satisfaction subscale (dependent variable)

**Fig. 2:**
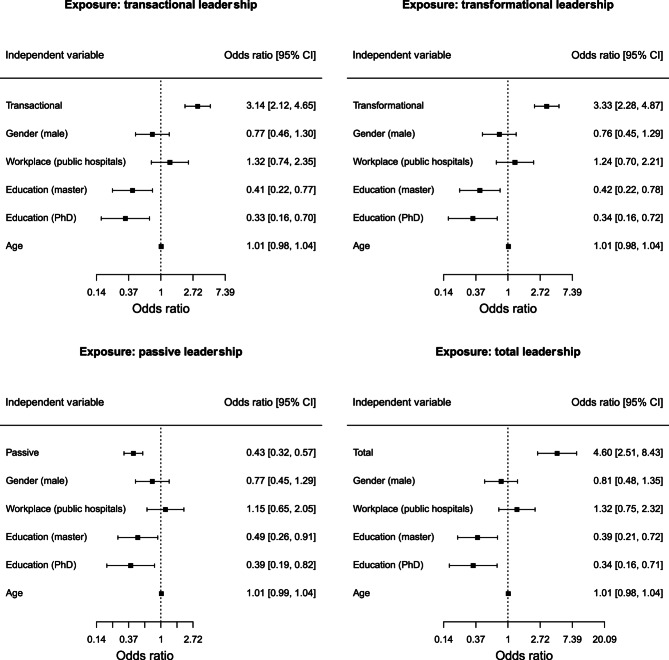
Odds ratios with 95% confidence intervals from logistic regression models for each pair between a leadership style (independent variable) and total job satisfaction (dependent variable). All models are adjusted by age, gender, education and workplace

In Fig. 1 we present the results [odds ratios with 95% Confidence Intervals (CI)] of different logistic regression models examining the association between leadership styles (independent variable) and job satisfaction subscales (dependent variables) among physicians. We found that transactional and transformational leaderships were positively associated with the nine subscales and the total scale of job satisfaction (i.e. odds ratios greater than 1), while passive leadership was negatively associated with job satisfaction. The total leadership was also positively associated with job satisfaction subscales, being statistically significant for six out of nine subscales. Total leadership was also statistically significantly associated in a positive manner with total job satisfaction [odds ratio = 3.88, 95% CI (2.27, 6.63)]. Finally, the above results persisted when examined by multivariable-adjusted regression models between each leadership style (independent variable) and total job satisfaction (dependent variable) after adjusted by gender, workplace, education and age (Fig. 2). Another interesting finding is that in the four models adjusted by potential confounders, we found that higher educational level (master or PhD) is negatively associated with total job satisfaction compared to lower educational level. We also show that the estimates for age, gender and workplace (public hospitals vs. health centers) are not statistically significant (Fig. 2). Even after performing sensitivity analyses using the median value of each satisfaction subscale as a cut-off point to dichotomise the subscales, the results did not differ from the main regression models (Fig. 1S, Table 2S and Fig. 2S in the Supplement).

## Discussion

In summary, we evaluated the association between physician managers’ leadership styles and job satisfaction among physicians in the public healthcare system of Cyprus. The results of our study demonstrated that transformational and transactional leaderships were strongly and positively associated with job satisfaction while passive leadership was characterized by a negative association. However, as an overall result of the above findings, the measure of total leadership was positively associated with job satisfaction among health professionals in the public health sector of Cyprus. It is also worth highlighting that both leadership styles (transformational and transactional) show similar strong positive associations with health professionals’ job satisfaction in the same subscales, namely, pay, promotion, supervision, fringe benefits, contingent rewards, communication and total satisfaction.

Several studies have described the positive contribution of physician managers using transformational leadership practices [27]. Weberg emphasized that a transformational leader can extend and elevate the interests of staff by facilitating employee commitment to the mission and values of the organization and could lead staff to rise above their personal interests [28]. Lavoie-Tremblay et al. indicated that transformational leadership potentially lead to high quality care and weak intention to quit the healthcare facilities and conversely, abusive leadership potentially lead to poorer quality care and strong intention to leave the organization due to lower job satisfaction [29]. Moreover, our findings are compatible with Kitsios et al. who considered job satisfaction as the overall emotional reaction of people to their job and reported that a professional-supportive leadership style in Greece may have a positive influence on job satisfaction. They also stated that by better understanding the above relationships, public health policy makers could intervene with appropriate incentives and programs that could positively affect employees’ job satisfaction among health professionals [30]. To this day, transformational leadership remains the better-known leadership approach [31], and our study findings are strongly compatible with the existing scientific literature on this topic as evaluated in Cyprus.

Our study has a relatively high response rate; despite the fact we had excluded a sizable number of participants due to missing values. Moreover, data were collected from physicians and no other healthcare workers in the public sector. The relatively small sample size does not allow for a more detailed statistical analysis between the different subscales of job satisfaction. Finally, in this study, we have examined the association of leadership with job satisfaction, although there are several other aspects of health professionals’ resource management styles that are also involved in this complex interconnection network that have not been addressed.

## Conclusion

We have found that transactional and transformational leadership styles were strongly and positively associated with health professionals’ job satisfaction in most subscales of the job satisfaction questionnaire used in this study. On the contrary, passive leadership style was associated with a negative impact on job satisfaction. Our study findings provide a strong insight regarding the association of leadership with several job satisfaction attributes.

Provided that our reported associations are based on underlying causal relationship, our study findings could support relevant policies and educational programs to promote transactional and transformational leadership styles among physicians’ managers in the public healthcare system of Cyprus. Such an approach is likely to increase health professional job satisfaction and enhance the retainment rate of health professionals in the public system along with supporting additional positive outcomes including the quality of healthcare services.

Our findings also support the important role of associated training to promote transactional and transformational leadership in the healthcare sector in preparing new leaders that could facilitate organization-wide changes during this highly demanding period of innovative technology and digital transformations in the healthcare sector.

## Supplementary Information

Supplementary Material 1: Table 1S: Pairwise Pearson correlations between the leadership subscales.

## Data Availability

Data Availability Statement: Data are available upon request.
