# Supplementary material for: Leadership and job satisfaction among physicians in the Cyprus public healthcare system
Source: BMC Health Serv Res. 2025 Aug 6;25:1032. doi: 10.1186/s12913-025-13241-3 (PMC12326732; doi:10.1186/s12913-025-13241-3)
Supplement: Supplementary file 1 — Supplementary Material 1: Table 1S: Pairwise Pearson correlations between the leadership subscales. [file 12913_2025_13241_MOESM1_ESM.docx]

Table 1S: Pairwise Pearson correlations between the leadership subscales

|  | transformational | transactional | passive | total |
| --- | --- | --- | --- | --- |
| transformational | 1 | 0.82 | -0.60 | 0.91 |
| transactional | 0.82 | 1 | -0.45 | 0.88 |
| passive | -0.60 | -0.45 | 1 | -0.24 |
| total | 0.91 | 0.88 | -0.24 | 1 |
